# Treatment of severe pressure ulcers with protein-enriched filtered platelet-rich plasma (PEF_PRP_): a possible management

**DOI:** 10.3389/fbioe.2023.1279149

**Published:** 2024-01-15

**Authors:** Laura Mazzucco, Valeria Balbo, Enrico Maria Zingarelli, Manuela Desilvestri, Manuela Marchioni, Luca Perrero, Francesca Pollis, Ilaria Varvello

**Affiliations:** ^1^ Transfusion Medicine and Regeneration Medicine, Azienda Ospedaliera SS Antonio e Biagio e Cesare Arrigo, Alessandria, Italy; ^2^ Department of Plastic and Reconstructive Surgery, Azienda Ospedaliera SS Antonio e Biagio e Cesare Arrigo, Alessandria, Italy; ^3^ Neuro-Rehabilitation Unit, Rehabilitation Department, Azienda Ospedaliera Nazionale SS Antonio e Biagio e Cesare Arrigo-Alessandria, Alessandria, Italy

**Keywords:** pressure ulcers, dressing, plasma proteins, protein-enriched filtered platelet-rich plasma (PEF_PRP_), neurologic patient

## Abstract

**Background:** Biological dressings with non-transfusion blood components are among the treatments available for pressure ulcers (PUs). Biological dressings contain active concentrated pro-regenerative molecules that can modify and switch off local inflammatory pathways. This re-establishes the physiological homing, which results in healing. In our study, we used a biological component obtained by ultrafiltration of plasma-platelet concentrate: protein-enriched filtered platelet-rich plasma (PEF_PRP_) with a higher platelet and higher plasma protein concentration. We tested whether treatment with PEF_PRP_ could improve healing in advanced-stage pressure ulcers with a large surface area. All the patients in this study had a surgical indication but were not able to undergo surgery for various reasons.

**Materials and methods:** Ten patients with severe neurological disability and advanced-stage sacral pressure ulcers were treated with allogenic PEF_PRP_. The mean lesion surface area at T0 was 13.4 cm^2^ ( ± 9.8 SD). PEF_PRP_ was derived from allogenic plasma-platelet apheresis that had been pre-ultrafiltered with a ProSmart™ filter (Medica, Italy) to obtain a concentration after filtration of the plasma protein (12–16 g/dL) and platelet (1–1.2 x 10^6^ microL).

**Results and Conclusion:** All cases showed a reduction in the surface area of the pressure ulcer and in the Pressure Ulcer Scale for Healing (PUSH) score. The mean reduction values at week 6 were as follows: −52% for surface area and −21% for PUSH. Rapid wound healing is fundamental to avoid infections and improve patients’ quality of life. This blood component builds new tissue by creating a new extracellular matrix. This, in turn, promotes rapid restoration of the three-dimensional structure of the tissue necessary for healing deeper wounds. PEF_PRP_ shrinks the PU and improves its morphological features (reducing undermining and boosting granulation tissue). PEF_PRP_ also promotes tissue restoration, obtaining an optimal scar. It is a safe and feasible treatment, and these preliminary results support the use of PEF_PRP_ in the treatment of pressure ulcers. PEF_PRP_ dressings could be integrated in the standard treatment of advanced-stage PU.

## 1 Introduction

Pressure ulcers (PUs) are localized skin injuries usually found on bony prominences. They may occur in the superficial skin or in the underlying tissue. PUs are caused by hypoxic damage due to pressure and/or shear stress on the skin. They represent a medical, social, and economic burden that significantly affects the patient’s condition and the rehabilitation programs. The treatment of PUs is expensive and requires multidisciplinary management (Hill et al., 2022). The most common risk factors for the development of PUs are extrinsic and intrinsic hypoxemia (e.g., poor mobilization, pressure or shear force, smoking, and COPD), changes in sensitivity, poor nutrition, urinary and fecal incontinence, and overall patient status (age, hematological diseases, infectious diseases, and cognitive deficiency). The NPUAP/EPUAP classification stages the severity of the lesion and defines the treatment for each stage (Jan et al., 2019). Nutritional status in subjects at risk and in those with PUs must be monitored; in fact, poorly nourished patients often show a major risk of developing PUs and a delay in the healing process (Saghaleini et al., 2018; Munoz and Posthauer, 2022). Patient mobilization is fundamental for the treatment and prevention of PUs; weight distribution and the use of high skin protection mattresses and cushions help improve comfort and hygiene (Shi et al., 2021). PU treatment consists of debridement, cleaning, treatment of underlying infection (if needed), and dressing. In clinical practice, we often use dressings containing hydrocolloid, hydrogel, calcium alginate, and foam. If these are not effective, and the PU worsens (with new formation of necrotic tissue, tunneling, and undermining), physical therapies (negative pressure and pulsed electrical stimulation) and surgery are other options (Moore and Patton, 2019). Biological dressing is also an available treatment for PUs, specifically dressings containing non-transfusion blood components (platelet gel) (Aprili et al., 2013). Chronic ulcers of different etiologies have in common a prolonged inflammatory response. It is possible to halt this process by providing platelet-derived growth factors (GFs) and plasma proteins to the ulcer to facilitate the tissue healing process (Qu et al., 2021; Singh et al., 2021). Ten patients with large surface-area PUs were treated with protein-enriched filtered platelet-rich plasma (PEF_PRP_) instead of surgery (Mazzucco et al., 2021). Surgery is the elective treatment for lesions at this stage, but where elective surgical treatment is not possible, whether on medical grounds or due to patient refusal, an alternative is needed.

The EPUAP 2019 guidelines suggest treatment with platelet gel for advanced-stage PUs (grade of recommendation: B1); the rational biological use of topical blood components is to modify and switch off the local inflammatory response by providing concentrates of regenerating active molecules (GFs, anti-inflammatory interleukins, fibrin, and extracellular matrix proteins) (Olczyk et al., 2014). This would lead to healing of the lesion through the stimulation of cellular physiological homing and angiogenesis (Anitua et al., 2012; Collins et al., 2021). For this study, we used PEF_PRP_ obtained from the ultrafiltration of allogenic blood components (plasma-platelet apheresis) that contains five times the platelet concentration compared to the baseline, that is not aggressive on platelets that preserve their structure and function, and that has a high plasma protein concentration (approximately two times the usual concentration). PEF_PRP_ collects and concentrates platelets and all the proteins present in the plasma and released by platelets (exosomal component and other), ensuring the complete and total collection of all PRP molecules that are normally lost during centrifugation and preparation by other methods (Dhurat and Sukesh, 2014; Everts et al., 2020). The purpose was to provide GFs derived from concentrated platelets to stimulate cells and plasma proteins to regenerate the extracellular matrix. Allogenic blood components were chosen (Van der Bijl et al., 2019; Liao et al., 2020; Akbarzadeh et al., 2021; Saputro et al., 2022); we collected the plasma-platelet apheresis from the donor to have more products available for treatment (large surface lesions) and have a more standardized equivalent product for all the patients enrolled in the study (treatment period 8 weeks). In addition, the PEF_PRP_ was ready for use, without having to collect blood from debilitated patients for each treatment. The purpose of this study was to determine the safety and feasibility of treating PUs with PEF_PRP_ as an alternative to plastic surgery. In PEF_PRP_, there are 30 upregulated proteins that are involved in cytoskeleton organization, regulation of proteolysis, and cellular response to cytokine stimulus. These proteins were classified by their function according to keywords related to the healing process of damaged tissues and corresponding to pro-inflammation, anti-inflammation, wound healing, clot stabilization, and anti-microbial and other plasma components such as complement system, cell–matrix adhesion, and immunoglobulins (Piccin et al., 2017; Cecerska-Heryć et al., 2022). The clot stabilization and cell–matrix adhesion proteins (fibrinogen, fibrin, fibronectin, and thrombin) contribute to a three-dimensional environment that is a crucial condition for driving cell–cell and protein–protein interactions and achieving tissue regeneration and healing. PEF_PRP_ is a new topical hemocomponent with multiple functions since it provides molecules stimulating biological mechanisms of resident cells and reconstruction of the extracellular matrix.

Our previous studies had shown that patients with chronic non-healing wounds showed a substantial improvement when treated with the PLT gel in an average time of 10 weeks (Mazzucco et al., 2004); one of the purposes of the study was to evaluate if the treatment with PEF_PRP_ improved the healing time frame (8 weeks) (Wallace et al., 2023) even on large wounds. Eight weeks was considered a sufficient time to assess reduction in wound size and/or possible complications. Reduction in lesion area, with particular attention to physical and morphological characteristics such as exudate and basal tissue (epithelial or granulation tissue, slough, and necrosis), was the principal indicator of the study output.

## 2 Materials and methods

For this prospective study, 10 patients (limited number due to difficulties in enrollment because of restrictive inclusion criteria including non-acceptance/non-eligibility to the surgical approach) were enrolled in the Neurorehabilitation Department of the Physical Medicine and Rehabilitation—Hospital SS. Antonio e Biagio di Alessandria (Piedmont); the patients had large pressure ulcers, which were already treated for at least 6 weeks with conventional dressings without result. The study took 1 year for enrollment (the study was postponed due to COVID-19).

Patients selected for treatment were all candidates for reconstructive surgery but not feasible because some were at risk of surgical complications or were non-compliant (refusal of surgery). The mean age of the patients was 65 years (SD ± 9.67). The study was conducted in accordance with the Helsinki Declaration and was approved by the local ethics committee (prot.ASO19.07CE 2019). The subjects were given a detailed explanation about the study and signed three informed consent forms (one to use allogenic blood components, one for the data collection, and one for the privacy policy). The study enrollment included a medical examination carried out by a specialist in physical medicine and rehabilitation, an evaluation of the PU, and staging (EPUAP) carried out by a plastic surgeon. Each patient showed advanced-stage sacral PUs (8 subjects: stage IV and 2 subjects: stage III). The patients presented with neurological disability with different etiologies: three severe brain injuries, two spinal cord injuries, and five critical illness polyneuropathies. Admission assessment scales were issued by the rehabilitation specialist. The mean Barthel Index (BI) was 3.2 ( ± 3.8 SD—BI from 0 to 20), and the mean Functional Independence Measure (FIM) was 45.4 ( ± 21.7 SD—FIM from 18 to 126). At the beginning of the study, the patients were clinically stable (mean hemoglobin 11.7 mg/dL ± 1, 5 SD—mean WBC 7862/mm^3^ +/− 2379 SD) and had negative SARS-CoV-2 swabs.

### 2.1 Nutritional support and high skin protection surfaces

The nutritional status was evaluated during hospitalization. We monitored the serum protein and albumin levels, and nutritional counseling was given to ensure adequate protein intake (in accordance with EPUAP guidelines). Optimal skin protection mattresses and cushions were provided to reduce pressure and shear stress on the skin.

### 2.2 Preparation and treatment with protein-enriched filtered platelet-rich plasma (PEF_PRP_)

**FIGURE 1:**
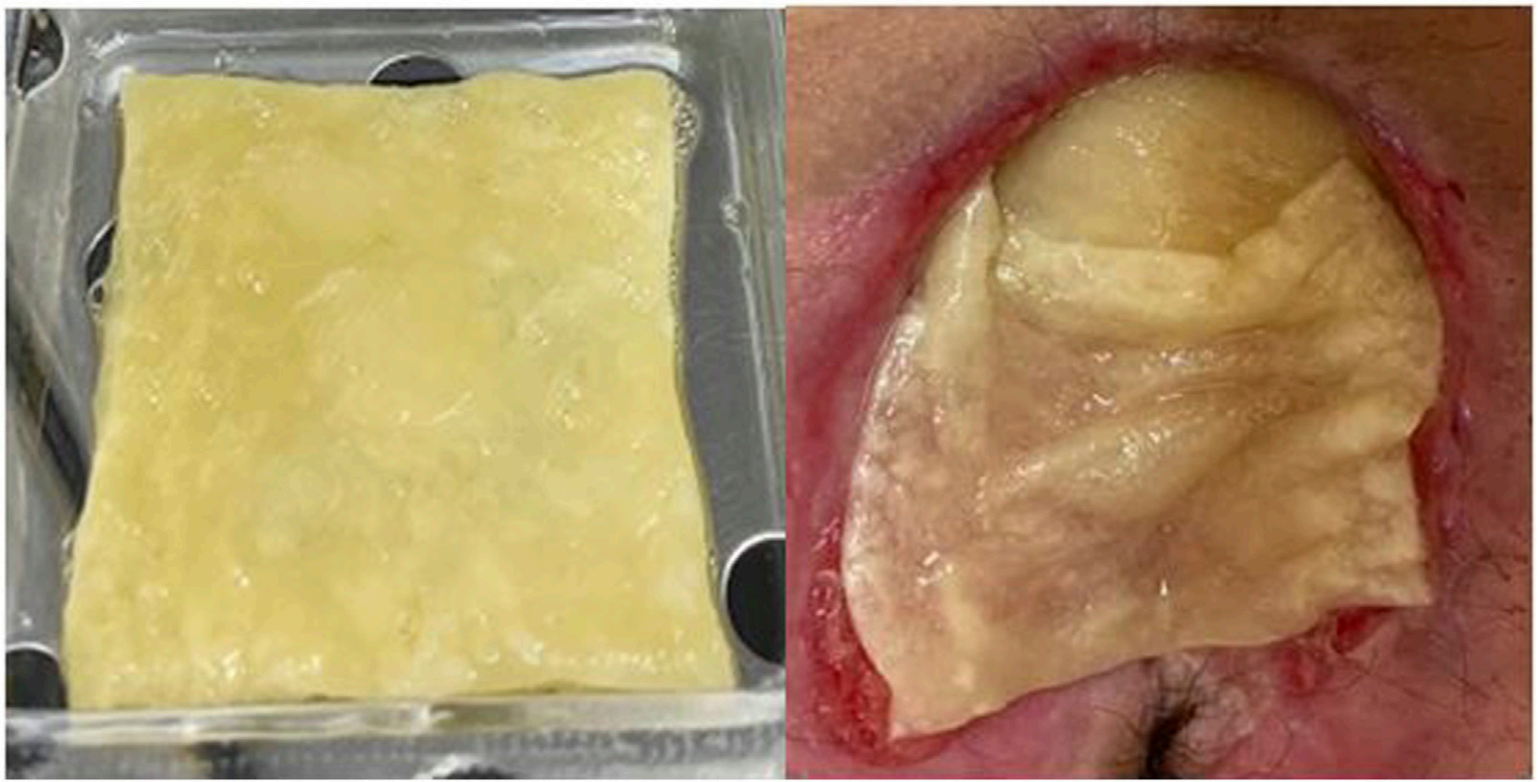
Biological dressing: matrix with PEF_PRP_ and at the site of the pressure ulcer.

PEF_PRP_ was derived from plasma-platelet apheresis collected at the Blood Transfusion Center, Alessandria Hospital, Italy. This blood component was obtained according to standard transfusion procedures (DM 2 November 2015—Italian Law). The platelet concentrate was standardized at a concentration of 1 × 10^6^/microL with plasma that had been prefiltered using a ProtSmart™ filter (Medica SpA, Medolla, Italy). The plasma protein concentration after filtration was between 12 and 16 g/dL. The PEF_PRP_ obtained was divided into 8-mL samples and stored in blood bank refrigerators at a temperature of −40°C. Type I collagen and hyaluronic acid (Fidia Farmaceutici S.p.A,, Italy) matrices were used to spread and stabilize the product over the lesion. The biological dressing with PEF_PRP_ was applied to infection-free lesions aiming to cover most of the PU surface (Figure 1) and was reapplied every 3 days, and the treatment lasted for 8 weeks (56 days).

### 2.3 Surface evaluation and statistical analysis

Before PEF_PRP_ treatment, all the PUs were surgically debrided to remove necrotic tissue. After this procedure, they were staged. The bed of the PUs was evaluated for local bacterial infection (GRAM+ and GRAM-) with fluorescent light technology (MolecuLight Smith and Nephew, Canada). Photographs were taken to provide evidence of changes on the lesion surface; the size of the lesion was measured with a digital program (ImageJ.exe). The data were analyzed with Minitab. The Pressure Ulcer Scale for Healing (PUSH tool—NPUAP) was used for assessment and monitoring. PUSH is a widely used tool developed by the National Pressure Ulcer Advisory Panel (NPUAP) that grades pressure ulcers based on wound size, wound bed tissue type, and exudate amount. PUSH and the surface area were measured weekly until the end of the treatment. The data obtained were analyzed using descriptive statistics, and the results were expressed as sums or percentages (Thomas et al., 1997; Stotts et al., 2001; Pressure Ulcer Scale for Healing PUSH, 2013; Choi et al., 2016; Rennie et al., 2019).

## 3 Results

**FIGURE 2:**
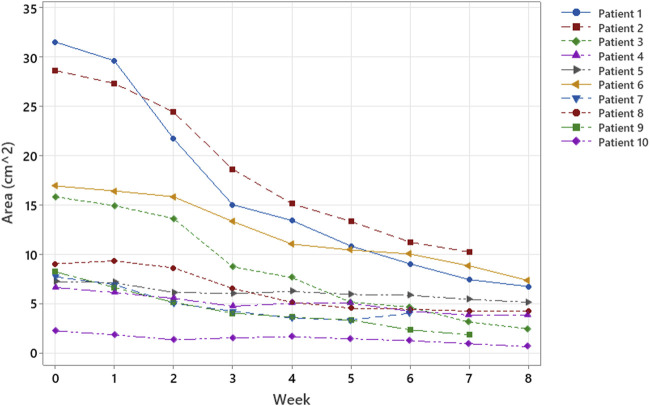
Trend of the surfaces of the areas under investigation over time. All wound areas reduced; four patients had a more abrupt reduction in the first 3–4 weeks.

**FIGURE 3:**
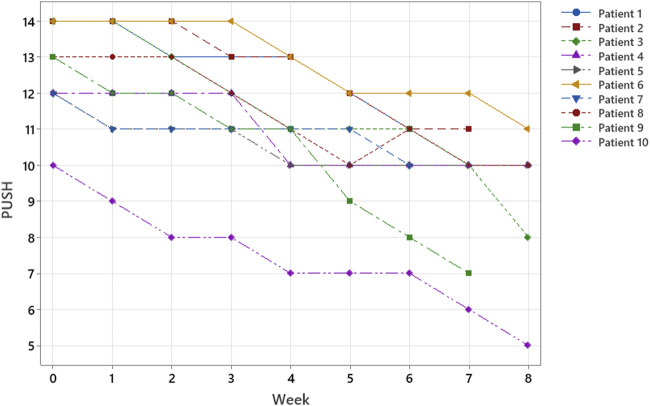
According to ulcer classification (surface, exudate, and wound tissue type)—PUSH SCORE; within 8 weeks, all patients responded to treatment, and three patients healed in only 6–7 weeks.

**FIGURE 4:**
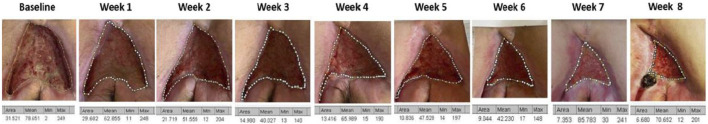
Representative images of the results (case 1).

Of the 10 patients evaluated, 7 were treated for 8 weeks (56 days), 2 for 7 weeks (49 days), and 1 for 6 weeks (42 days). At T0, the mean lesion superficial area was 13.4 cm^2^ ( ± 9.8 SD), and the mean PUSH was 12.8 ( ± 1.3 SD). All cases showed a reduction in the PU surface area (Figure 2) and PUSH (Figure 3). The mean reduction at week 6 was −52% for the surface area and −21% for PUSH. For the statistical analysis, we considered that consistency in our sample was obtained at Week 6. In the patients treated for 7 or 8 weeks, further reductions in surface area and PUSH were obtained (mean reduction in wound surface area: 13.4–3 cm^2^). None of the patients suffered any side effects. At T6, the mean lesion surface area was 5.7 cm^2^ ( ± 3.3 SD), and the mean PUSH was 10.1 ( ± 1.5 SD). At discharge from the neurorehabilitation department, the assessment scales showed a mean Barthel Index score of 11.8 ± SD 6.2 and a mean FIM of 89.3 ± 33.2 (Table 1; Figure 4).

**TABLE 1:** Clinical results over time intervals (PUSH: Pressure Ulcer Scale for Healing).

		T0	T1	T2	T3	T4	T5	T6	T7	T8
Case 1	Area (cm^2^)	31.5	29.6	21.7	15.0	13.4	10.8	9.0	7.4	6.7
	PUSH	14	14	13	13	13	12	11	10	10
Case 2	Area (cm^2^)	28.6	27.3	24.4	18.6	15.1	13.3	11.2	10.2	—
	PUSH	14	14	14	13	13	12	11	11	—
Case 3	Area (cm^2^)	15.8	14.9	13.6	8.7	7.6	5.1	4.6	3.1	2.4
	PUSH	14	14	13	12	11	11	11	10	8
Case 4	Area (cm^2^)	6.6	6.1	5.5	4.7	5.0	5.0	4.2	3.8	3.8
	PUSH	12	12	12	12	10	10	10	10	10
Case 5	Area (cm^2^)	7.2	7.1	6.1	6.0	6.2	5.9	5.8	5.4	5.1
	PUSH	12	11	11	11	10	10	10	10	10
Case 6	Area (cm^2^)	16.9	16.4	15.8	13.3	11.0	10.4	10.0	8.8	7.3
	PUSH	14	14	14	14	13	12	12	12	11
Case 7	Area (cm^2^)	7.7	6.9	5.0	4.2	3.5	3.3	4.0	—	—
	PUSH	12	11	11	11	11	11	10	—	—
Case 8	Area (cm^2^)	9.0	9.3	8.6	6.5	5.1	4.5	4.4	4.2	4.2
	PUSH	13	13	13	12	11	10	11	10	10
Case 9	Area (cm^2^)	8.2	6.6	5.1	4.0	3.6	3.3	2.3	1.8	—
	PUSH	13	12	12	11	11	9	8	7	—
Case 10	Area (cm^2^)	2.2	1.8	1.3	1.5	1.6	1.4	1.2	0.9	0.6
	PUSH	10	9	8	8	7	7	7	6	5

## 4 Discussion

Timing in PU healing is fundamental to avoid infections and improve the patient’s quality of life. It has been known for many years that regenerative medicine protocols involving treatment with blood components can stimulate and accelerate wound healing (Greppi et al., 2011). The etiology of PU is varied, but local hypoxic damage plays a fundamental role in its development (NPUAP). Ischemia and hypoxia due to various causes reduce the supply of nutrients to the tissues, both locally and systemically. Providing concentrates of biomolecules derived from donors to the site of the lesion can boost biological pathways that have become blocked by *in situ* catabolites and inflammatory molecules. Previous studies have demonstrated that advanced wound therapy using local applications of PRP is a promising alternative to standard saline dressings in PU healing; in young patients, there is a significant improvement in the histopathological condition of the ulcer after 5 weeks (Singh et al., 2014; Jharwal Rajesh et al., 2023). Conceptually, PEF_PRP_ is a new product derived from the ultrafiltration of platelets and plasma concentrates. It is rich not only in platelets—whose key components for the regeneration or replacement of tissue are GFs (VEGF, EGF, and PDGF-bb)—but also in plasma proteins, which play a role in dermal regeneration. This characteristic promotes the rebuilding of the extracellular matrix, which is the 3D scaffold for cells. This new environment is proangiogenic for resident stem cells and induces the development of endothelial sprouts, their transformation into vessels, and the final maturation of the capillary network into granulation tissue.

Given that the study has some limitations, that is, the low number of subjects enrolled and non-homogeneity in terms of age, comorbidity, and lesion site, this approach was chosen to overcome the conditions of the unsuitability of the patients for surgical treatment and the need to induce rapid stimulation for closure of the large lesion. Our results show a mean reduction of 52% in lesion area and 21% in PUSH after 6 weeks of treatment (Table 1). All cases showed improvement, although two showed only minimal improvement and a slight reduction in the surface area. Even the evidence of no worsening is a positive result, considering that these are patients with tissue hypoxia of various etiologies. Choosing this treatment over surgery has the advantage that it spares the patient’s anatomy. Surgical transfer of muscle flaps from the *gluteus maximus* or hamstrings invariably causes loss of body function, such as loss of strength in lower limb extension, which can lead to gait deficiency in ambulant patients (Kuo et al., 2014). It must be said that PU is more common in people with mobility deficiencies, such as spinal cord injury (SCI) patients who are wheelchair-bound. For this population, the availability of effective conservative treatment options is highly beneficial. Muscle tissue is limited and should be spared unless there is no other option available. Furthermore, surgery for sacral PU requires at least 30 days of bed rest in the prone position to allow the wound to heal. This requires increased healthcare assistance and longer hospitalization times, as well as the suspension of ongoing physiotherapeutic rehabilitation programs. Avoiding surgery also prevents possible surgical complications (hemorrhage, wound infection, and hematoma) and discomfort (local pain and prolonged immobilization) (Sameem et al., 2012). It is also clear that choosing a biological treatment to treat these wounds will reduce costs for the healthcare system (Demarre et al., 2015; Hajhosseini et al., 2020). However, this regenerative treatment is not always applicable; with advanced-stage PU and extensive destruction of the underlying tissue, the possibility of achieving proper healing without healthy tissue grafting is unlikely. Treatment with PEF_PRP_ requires a lot of time and compliance with care to achieve partial or total closure of the injury, but it is a matter of evidence that this product stimulates the formation of new tissue, ensuring also a better rooting of a possible graft to permanently close the lesion. A good option might be to integrate both treatments, e.g., reducing the PU using biological components and then performing surgery on the resulting smaller lesion. PEF_PRP_ could boost wound healing, and after a fixed timing of biological treatment, PU treatment could be reconsidered in favor of other therapies (e.g., standard dressing or surgery).

In conclusion, the combination of concentrated plasma proteins and platelet proteins may facilitate tissue regeneration, demonstrating that PEF_PRP_ is a safe and feasible treatment for PU. Moreover, PEF_PRP_ reduces the size of the PU and improves its morphological features: e.g., it quickly reduces undermining and improves physical features, promoting the formation of granulation tissue. PEF_PRP_ dressings could be integrated into the care of advanced-stage PU as a possible choice of natural biological treatment. These dressings could be used as conservative treatment alone or together with reconstructive surgery, though further studies are needed to confirm the efficacy of the latter.

## Data Availability

The original contributions presented in the study are included in the article/Supplementary Material; further inquiries can be directed to the corresponding author.
